# From Patient to Molecule: In Pursuit of Universal Treatments for TB

**DOI:** 10.1111/cts.12718

**Published:** 2019-11-29

**Authors:** Penny M. Heaton, Jeffrey S. Barrett

**Affiliations:** ^1^ The Bill & Melinda Gates Medical Research Institute Cambridge Massachusetts USA

Infectious diseases that are found primarily in the history books of high‐income countries are still leading causes of mortality in low‐income and middle‐income countries. A new model to develop interventional products against these diseases with limited market potential is needed. The Bill & Melinda Gates Medical Research Institute was created as a non‐profit biotech to develop interventional products against diseases that disproportionately affect the poor. This paper describes some of the institute's work on candidate drugs and vaccines against tuberculosis, the leading infectious diseases killer in the world.

## Gates Medical Research Institute: Mission and Approach

The Bill & Melinda Gates Medical Research Institute (Gates MRI) is a nonprofit biotechnology organization focused on diseases that disproportionately affect the poor. Our mission is to develop products that will help end the tuberculosis (TB) epidemic, eradicate malaria, end diarrheal diseases death in children, and reduce adverse maternal and newborn outcomes. We are focused on these disease areas because taken together they are responsible for 10 deaths every minute.

We believe we should utilize the same cutting‐edge technologies and development approaches for diseases prevalent in low‐income countries as those used for diseases of the rich world. Established in January 2018, the Gates MRI includes results‐oriented experts in product development with a special focus on areas previously well‐known to be associated with bottlenecks in product development, including individuals with expertise in bridging between discovery and development, manufacturing process development, systems biology, immuno‐assays, and quantitative sciences. The highest priority of the institute is the fight against TB, including development of TB vaccines and drugs.

## Tuberculosis Facts

TB, which is caused by the bacterium *Mycobacterium tuberculosis* (Mtb), primarily manifests as severe pulmonary disease. Nearly one quarter of the world's population is infected with 10 million new cases per year.^1^ Of those infected, 90% remain asymptomatic but 10% progress to severe pulmonary disease, claiming nearly 2 million lives each year.

TB is difficult to treat. Even uncomplicated disease requires a regimen of four drugs (isoniazid, rifampin, pyrazinamide, and either ethambutol or streptomycin) all of which are given daily for 2 months and two of which (isoniazid and rifampin) must be taken for an additional 4 months.^2^ Multidrug resistant TB, increasing in prevalence, can require up to seven drugs for 9 to 24 months. There is a single vaccine to prevent TB, the Bacille Calmette Guerin (BCG) vaccine, which is nearing its 100th birthday. It is indicated for infants and toddlers to prevent disseminated TB. The efficacy of the vaccine varies by population and is generally accepted to be ~ 50%.^3^ However, in low‐income countries, the population most severely affected are older adolescents and adults, those in the work force with young families to support.^4^ In addition, their only option if they develop TB is to take the complicated and often unaffordable drug regimens previously described. Can we not provide a better alternative, a regimen that is easier to take, easier to access, and affordable? Or better yet, provide a vaccine that prevents TB in the first place?

## Possible Alternatives to the Status Quo: TB Vaccines

A vaccine to prevent Mtb infection and stop the disease from developing in the first place would be the ideal tool for the young mothers and fathers in low‐income countries to help end the TB epidemic. For the one licensed TB vaccine that exists, BCG, studies evaluating the effectiveness of a booster dose in older adolescents and adults have shown conflicting results. Further, despite the fact that this is the most widely used vaccine in the world, there are significant gaps in scientific knowledge. We do not understand: (i) the mechanism by which it provides immunity against TB; (ii) if the current dose is the optimal dose; (iii) the best way to measure the dose level in the vaccine vial; and (iv) whether a booster given in adolescents/young adults can help prevent infection and pulmonary TB.

These gaps exist for many reasons. Mtb is an organism that has co‐evolved with humans for thousands of years, learning to evade the immune system in most of the hosts it infects. The immunologic mechanism of protection does not seem to be antibody, as is the case with most vaccines, thus leaving no marker by which dosing could be optimized (recall with vaccine development, dose is determined indirectly by the immune response vs. directly measuring the concentration in the blood). Further, once TB was well‐controlled in high‐income countries in the first half of the last century, additional resources to continue studying BCG or develop new vaccines were limited at best.

Building on the great work of other entities, including Aeras, TB Alliance, and The Bill & Melinda Gates Foundation (Gates Foundation), Gates MRI will try to answer some of these questions in a clinical trial targeted to begin in October 2019. Adolescents and young adults who received BCG at birth will be randomized to receive either a booster dose of BCG or placebo and followed for development of infection with Mtb. Immune response to the vaccine and to natural infection will be interrogated at multiple time points using state‐of‐the‐art assays that will measure not only antibody but also other cellular responses down to the level of a single cell. Using clinical trial and bio‐banked samples from other studies, it is hoped that the responses in protected and unprotected individuals can be distinguished and models built to predict whether BCG will be effective in other populations beyond infants and whether new TB vaccines can be expected to protect against Mtb.

## Possible Alternatives to the Status Quo: TB Drugs

Although the search continues for a better TB vaccine for older adolescents and adults, the next best thing would be a shorter, simpler, affordable drug regimen with minimal side effects. The most recently developed drug in the current, standard four drug‐regimen was approved in 1968.^5^ Yes, this is not a typographical error, it was approved over 50 years ago. Only three new TB drugs have been approved since, new entities for multidrug‐resistant TB approved in 2012, 2014, and 2019.^6^ Because of the low prevalence of TB disease in high‐income countries, the pipeline for new TB drugs has been very thin.

To address this, the Gates Foundation and several other collaborators formed the TB Drug Accelerator (TBDA) in 2012. This collaboration, which now includes 8 private sector partners and 10 research institutions, was created to screen existing compound libraries for activity against Mtb and catalyze identification of new targets. The effort has been successful; several preclinical candidates, including some with novel mechanisms of action, have been identified for potential clinical development.

**Figure 1:**
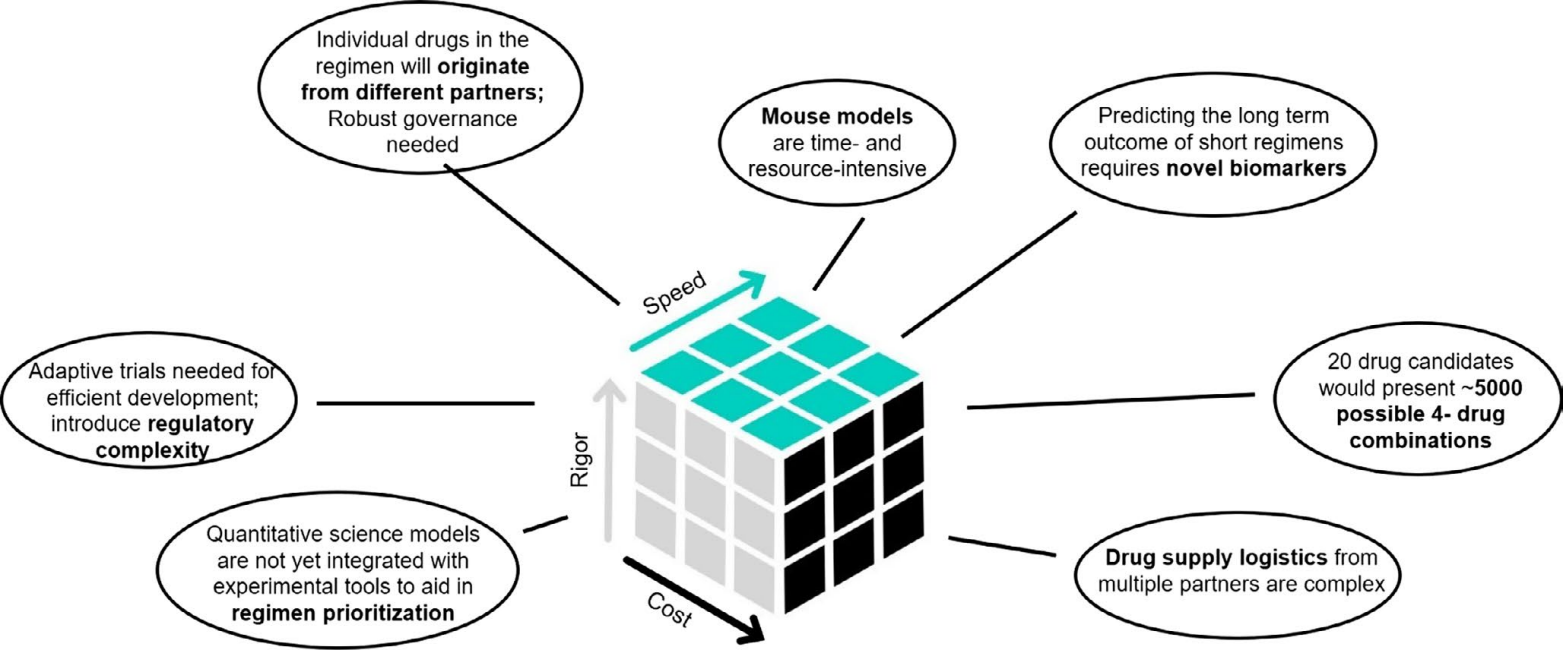
Challenges in global health tuberculosis drug regimen development.

Although exciting, moving novel TB drug regimens into clinical development is not without its challenges (**Figure** 1). The individual drugs needed for an optimal regimen are likely to originate from different organizations. Regimen selection and down‐selection will be challenging; for example, 20 preclinical candidates represent ~ 5,000 different possible four‐drug regimens. Preclinical animal models are not ideal; the preferred mouse model is time and resource‐intensive, has yet to be optimized for regimens, and does not consistently predict clinical outcomes. Novel biomarkers are needed to predict the long‐term outcome of short regimens. Finally, quantitative science models are not yet integrated with experimental tools to aid in regimen prioritization.

The Gates MRI is working to understand the data gaps, conduct experiments to fill in those gaps, and move forward with a model‐informed drug development approach consistent with the way in which drug development is conducted for high‐income countries.^6^ We are working with partners on a preclinical “funnel” for downselection of regimens for progression into clinical development. The conceptual funnel is, of course, a combination of preclinical experiments and quantitative models and tools that both refine the unique experimental conditions and optimize the experimental design, as well as provide a means to challenge the physiologic constraints of the individual experimental models (*in vitro* and *in vivo*). For instance, the relapsing mouse model is being adapted as a starting point to evaluate regimens vs. single drugs with a simple rank‐order evaluation. Complimentary to the relapsing mouse model and hollow fiber, *in vitro* experiments are predictive and mechanistic models operationalized to better understand drug synergies of different combinations as the next level of quantitation beyond simple rank ordering (response surface modeling approach). The Gates MRI, in conjunction with collaborators in the global health ecosystem, is also supporting a quantitative systems pharmacology model, which fuses existing and developing experimental and quantitative model assets to inform new biomarker strategies, evaluate patient phenotypes, which may preferentially respond to certain combinations, and support clinical dosing recommendations.^7^ Preclinical models with high fidelity will serve as the basis for clinical trial simulations that will precede actual human phase testing.

**Figure 2:**
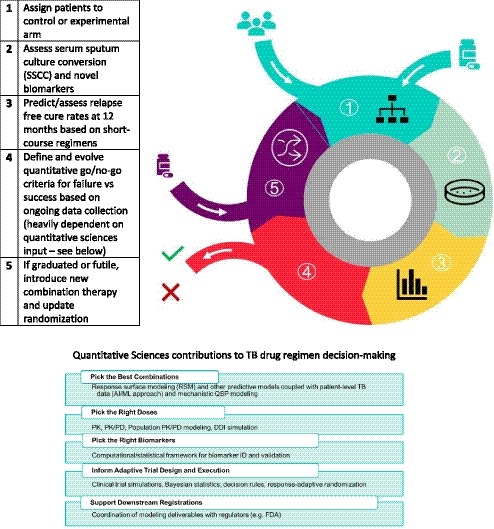
The Bill & Melinda Gates Medical Research Institute approach to adaptive platform trial design to support tuberculosis drug regimen development. AI/ML, artificial intelligence/machine learning; DDI, drug‐drug interaction; FDA, US Food and Drug Administration; PK, pharmacokinetics; PK/PD, pharmacokinetic‐pharmacodynamic; QSP, quantitative systems pharmacology.

Early examples of Gates MRI's investment in the model‐informed drug development (MIDD) approach include physiologically‐based pharmacokinetic modeling to inform first‐in‐human dose selection for a phase I trial, population‐pharmacokinetic modeling to inform sampling schemes for a phase II early bactericidal activity trial, and extensive clinical trial simulation to support sample size requirements, subject selection criteria, and evaluate the interferon‐gamma release assay threshold sensitivity on end point determination for our upcoming BCG revaccination trial. There is a clear roadmap^8^ for the model‐informed drug development approach laid out by our industrial^9^ and academic partners^8^ in collaboration with colleagues at the Gates Foundation. Our commitment is to adopt this roadmap with the guidance of global regulatory authorities. The ultimate vision is to move new regimens into a clinical trial platform for evaluation utilizing an adaptive study design and new biomarkers with sophisticated bioinformatics, iterating based on evolving data to identify improved regimens in as short a timeframe as possible—moving from molecule to patient and back again to fight this disease (**Figure** 2). During the process of evaluating and hopefully obtaining approval and recommendations for shorter and safer TB drug regimens, it is expected that tools developed along the way may serve as the basis for a personalized medicine strategy for patients with TB in the future.

In summary, although the BCG vaccine and the currently available drugs will have their rightful place in history in making an impact on TB, young people and families in low‐income countries deserve more than a century old vaccine and half a century old drugs to treat this disease. We believe we can utilize state‐of‐the‐art technologies and product development approaches of the modern era to finish the job, to bring us closer to the Sustainable Development Goal of a 90% reduction in TB mortality by 2030 toward ultimately ending the TB epidemic.

## Funding

The Bill & Melinda Gates Medical Research Institute is a fully funded subsidiary of the Bill & Melinda Gates Foundation.

## Conflict of Interest

Both authors declared no competing interests for this work.
